# Glutathione peroxidase 8 expression on cancer cells and cancer‐associated fibroblasts facilitates lung cancer metastasis

**DOI:** 10.1002/mco2.152

**Published:** 2022-08-10

**Authors:** Yu‐Lian Xu, Luo‐Wei Yuan, Xiao‐Ming Jiang, Min‐Xia Su, Mu‐Yang Huang, Yu‐Chi Chen, Le‐Le Zhang, Xiuping Chen, Hong Zhu, Jin‐Jian Lu

**Affiliations:** ^1^ State Key Laboratory of Quality Research in Chinese Medicine Institute of Chinese Medical Sciences University of Macau Macao China; ^2^ Zhejiang Province Key Laboratory of Anti‐Cancer Drug Research College of Pharmaceutical Sciences Zhejiang University Hangzhou China; ^3^ Department of Pharmaceutical Sciences Faculty of Health Sciences University of Macau Macao China; ^4^ MoE Frontiers Science Center for Precision Oncology University of Macau Macao China; ^5^ Guangdong‐Hong Kong‐Macau Joint Lab on Chinese Medicine and Immune Disease Research University of Macau Macao China

**Keywords:** CAF, focal adhesion, GPX8, lung cancer, metastasis

## Abstract

Lung cancer is the leading cause of cancer death worldwide, of which lung adenocarcinoma (LUAD) is the most common subtype. Metastasis is the major cause of poor prognosis and mortality for lung cancer patients, which urgently needs great efforts to be further explored. Herein, glutathione peroxidase 8 (GPX8) was identified as a novel potential pro‐metastatic gene in LUAD metastatic mice models from GEO database. GPX8 was highly expressed in tumor tissues, predicting poor prognosis in LUAD patients. Knockdown of GPX8 inhibited LUAD metastasis in vitro and in vivo, while it did not obviously affect tumor growth. Knockdown of GPX8 decreased the levels of p‐FAK and p‐Paxillin and disturbed the distribution of focal adhesion. Furthermore, GPX8 was overexpressed in cancer‐associated fibroblast (CAF) and associated with CAF infiltration in tumor microenvironment of lung cancer. GPX8 silence on fibroblasts suppressed lung cancer cell migration in the coculture system. BRD2 and RRD4 were the potential transcriptionally regulators for GPX8. Bromodomain extra‐terminal inhibitor JQ1 downregulated GPX8 expression and suppressed lung cancer cell migration. Our findings indicate that highly expressed GPX8 in lung cancer cells and fibroblasts functions as a pro‐metastatic factor in lung cancer. JQ1 is identified as a potential inhibitor against GPX8‐mediated lung cancer metastasis.

**Abbreviations**
BCA: bicinchoninic acid
BET: bromodomain and extra‐terminal
CAF: cancer‐associated fibroblast
CCL2: C‐C motif chemokine ligand 2
CDS: coding sequence
ECM: extracellular matrix
EGFR: epidermal growth factor receptor
ER: endoplasmic reticulum
GEO: Gene Expression Omnibus
GPX8: glutathione peroxidase 8
GSEA: Gene Set Enrichment Analysis
HE: hematoxylin eosin
HR: hazard ratio
HRP: horseradish peroxidase
IHC: immunohistochemical
IL6: interleukin 6
KEGG: Kyoto Encyclopedia of Genes and Genomes
KM: Kaplan–Meier
KRAS: kirsten rat sarcoma viral oncogene homolog
LUAD: lung adenocarcinoma
PBST: phosphate buffered solution with Tween‐20
PVDF: polyvinylidene fluoride
qPCR: quantitative real‐time polymerase chain reaction
RNA‐seq: RNA sequencing
SDS‐PAGE: sodium dodecyl sulfate polyacrylamide gel electrophoresis
shRNA: small hairpin RNAs
STR: short tandem repeat
TCGA: The Cancer Genome Atlas
TISCH: Tumor Immune Single‐cell Hub

## 1 INTRODUCTION

Lung cancer is one of the most prevalent malignant tumors with much high mortality and incidence rates around the world.^1^ Approximately, 85% of lung cancer belongs to non‐small cell lung cancer, which includes three main subtypes, adenocarcinoma, squamous cell carcinoma, and large cell carcinoma.^2^ Lung adenocarcinoma (LUAD) accounts for nearly 40% of all histological types of lung cancer with poor prognosis.^3^ It is quite common for lung cancer to metastasize before it is diagnosed in clinic. The preferential sites of lung cancer for metastasis are the brain, bones, liver, and adrenal glands.^4^ Osimertinib could cross the blood–brain barrier and was demonstrated efficacy for intracranial metastatic LUAD with epidermal growth factor receptor (EGFR) mutation,^5^ but the clinical outcomes for most metastatic LUAD remain poor. Tumor metastasis is still the major cause of cancer‐related death, leading to a relatively low five‐year survival rate in metastatic LUAD patients.^6^ In the process of metastasis, cancer cells spread from the primary site, go through the lymphatic system or blood vessels, and eventually develop new tumors in other tissues or organs of the body.^7^, ^8^ The pro‐metastatic molecular changes are quite important for cancer cells acquired metastatic self‐sufficiency. Thus, it is crucial to uncover more pro‐metastatic molecules for the development of prevention and treatment for metastatic LUAD. Great efforts have been made to explore the molecular mechanisms of LUAD metastasis and identify numerous pro‐metastatic factors as potential therapeutic targets in LUAD, such as CD109^9^ and ARNTL2,^10^ but there is still a long way to go for the clinical applications of these potential targets. Recently, more evidence suggested that tumor microenvironment (TME) is quite important for cancer progression and metastasis.^11^ Cancer‐associated fibroblasts (CAFs) are important for TME composition in lung cancer and involved in lung cancer metastasis through secretion of cytokines, growth factors, CAF‐specific proteins, and exosomes.^12^ Lung cancer metastasis is a multi‐step process with a variety of mechanisms, it urgently needs to further study the potential molecular mechanism of LUAD metastasis, providing new insights for the diagnosis, prevention, and treatment of metastatic LUAD.

Glutathione peroxidase 8 (GPX8) is a member of glutathione peroxidase family.^13^ The primary role of the GPX family is the modulation of peroxide accumulation, but GPX8 is a poorly characterized enzyme and its detailed function is largely unclear.^14^, ^15^ GPX8 is a membrane protein of the endoplasmic reticulum (ER) with a C‐terminal ER membrane localization signal, and its peroxidase activity could prevent the toxicity and leakage of Ero1α‐produced H_2_O_2_ in ER.^16^ Aside from the enzyme activity, the conserved *N*‐terminal transmembrane domain of GPX8 plays an essential role in the regulation of Ca^2+^ homeostasis and signaling.^17^ HIFα‐induced GPX8 expression could negatively control proliferative signaling under hypoxia and/or receptor tyrosine kinases (RTK) signaling conditions.^18^ Moreover, high expression of GPX8 is closely associated with poor prognosis in the patients with gastric cancer and promotes the progression of breast cancer and esophageal squamous cell carcinoma.^19^, ^20^, ^21^ However, the potential function of GPX8 in LUAD metastasis is largely unknown.

In this study, we identified GPX8 as a potential pro‐metastatic factor in lung cancer by in vitro and in vivo assays, which provides a new insight into the pathogenesis of lung cancer metastasis. High expression of GPX8 was associated with CAF infiltration in lung cancer. GPX8‐expressed in fibroblasts contributed to migration promotion in the coculture system. As BRD2 and BRD4 were validated as the upstream regulators of GPX8, inhibition of GPX8 by bromodomain and extra‐terminal (BET) inhibitors may serve as a novel therapeutic potential for lung cancer metastasis.

## 2 RESULTS

### 2.1 Glutathione peroxidase 8 overexpression is identified in the patients with LUAD and closely associated with tumor metastasis

**FIGURE 1:**
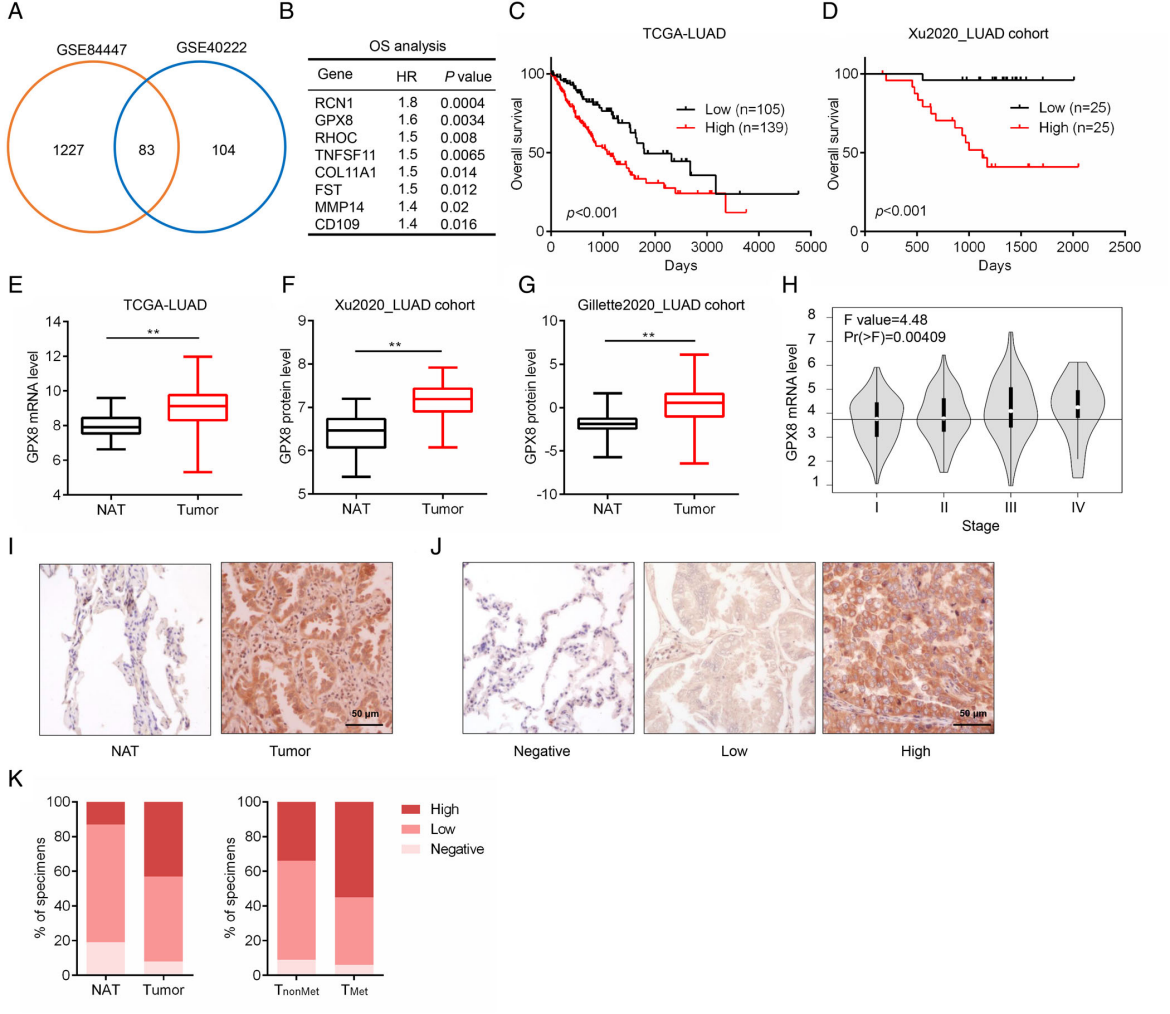
Glutathione peroxidase 8 (GPX8) overexpression is identified in the patients with lung adenocarcinoma (LUAD) and closely associated with tumor metastasis. (A) Venn diagram represented the overlap pro‐metastatic genes of the datasets (GSE8447 and GSE0222). (B) High expression of these genes identified from Venn diagram were associated with poor prognosis of LUAD patients ranked by hazard ratio (HR). (C) Kaplan–Meier (KM) survival curves were performed based on the TCGA database to show the overall survival of LUAD patients with high or low mRNA level of GPX8. (D) KM analysis of the overall survival based on the GPX8 protein expression at low and high levels in Xu2020_LUAD cohort. Statistical significance was determined by using log‐rank test. (E) The mRNA expression of GPX8 was compared between 515 LUAD tissues and 59 normal lung tissues from TCGA. (F) The protein expression of GPX8 was compared between 83 paired LUAD tissues and normal lung tissues from Xu2020_LUAD cohort. (G) The protein expression of GPX8 was compared between 101 paired LUAD tissues and normal lung tissues from Gillette2020_LUAD cohort. (H) The transcriptional level of GPX8 was compared between different stages of patients with LUAD in TCGA. (I) Representative images of the immunohistochemical (IHC) staining of GPX8 in paired normal tissues adjacent to the tumor (NAT) and tumor tissue from LUAD tissue microarray. Scale bar: 50 µm. (J) Representative images of the IHC staining of GPX8 from LUAD tissue microarray. Scale bar: 50 µm. (K) The distribution of samples according to the intensity of GPX8 IHC staining. NAT (*n* = 75) and tumor tissues (*n* = 75). Non‐metastasis tumor tissues (*n* = 44), metastasis tumor tissues (*n* = 31). *p < 0.05 and **p < 0.01

To identify the vital genes in promoting LUAD metastasis, two gene expression datasets of LUAD metastatic mice models (GSE84447 and GSE40222) were used for the analysis. Compared with non‐metastatic tumors, 83 overlapping genes were highly expressed (> 2‐fold changes) in metastatic tumors (Figure 1A). Since metastasis confers poor prognosis of LUAD patients,^22^ survival analysis of these genes was performed based on the mRNA level in LUAD patients from The Cancer Genome Atlas (TCGA). As a result, 8 genes (*RCN1*, *GPX8*, *RHOC*, *TNFSF11*, *COL11A1*, *FST*, *MMP14*, and *CD109*) were identified as the poor prognostic factors with significantly high hazard ratio in LUAD (Figure 1B). In addition to GPX8, the others have been reported to involve in cancer metastasis.^9^, ^23^, ^24^, ^25^, ^26^, ^27^, ^28^ RCN1 and COL11A1 are highly expressed in lung cancer and promote lung cancer cell proliferation, migration, and invasion.^29^, ^30^ RHOC is one of the three isoforms of the Rho family of GTPases and MMP14 is one of the matrix metalloproteinase family members, which are widely involved in cell movement.^25^, ^31^ TNFSF11, also named RANKL, is a critical regulator for cancer bone metastases.^32^ CD109 is reported as a potential diagnostic and therapeutic factor for lung cancer metastasis.^9^ The expression level of GPX8 was positively correlated with others in LUAD patients (Figure S1A–G). Moreover, both GPX8 mRNA expression and protein expression were negatively correlated with overall survival (Figure 1C,D) in TCGA_LUAD dataset and Xu2020_LUAD cohort. In addition, GPX8 was highly expressed in tumor tissues compared to the normal tissues both in the mRNA (Figure 1E) and protein levels (Xu2020_LUAD cohort and Gillette2020_LUAD cohort, Figure 1F,G). GPX8 is an enzyme and rarely expressed on blood cell and immune cells but high expressed in fibroblasts in lung (data not shown), which may be a good target for drug discovery. Its high expression in LUAD was independent of the main molecular subtypes *EGFR* and kirsten rat sarcoma viral oncogene homolog (*KRAS*) mutations (Figure S1H). Furthermore, the expression level of GPX8 in tumor tissues with the late stage of LUAD was much higher than that in the early stage (Figure 1H). The protein level of GPX8 in LUAD tissues microarray with 75 paired normal and tumor tissues was detected by immunohistochemical (IHC) staining. As shown in Figure 1I–K, GPX8 was highly expressed in the tumor tissues compared with the normal tissue adjacent to the tumor (NAT), and more GPX8‐high expressed tumors (55% vs. 34%) were observed in the metastasis group than that in the non‐metastasis group. These findings suggest that GPX8 is highly expressed in LUAD patients and its overexpression is associated with tumor metastasis and poor prognosis in the patients with LUAD.

### 2.2 Glutathione peroxidase 8 regulates migration and invasion in LUAD cell lines

**FIGURE 2:**
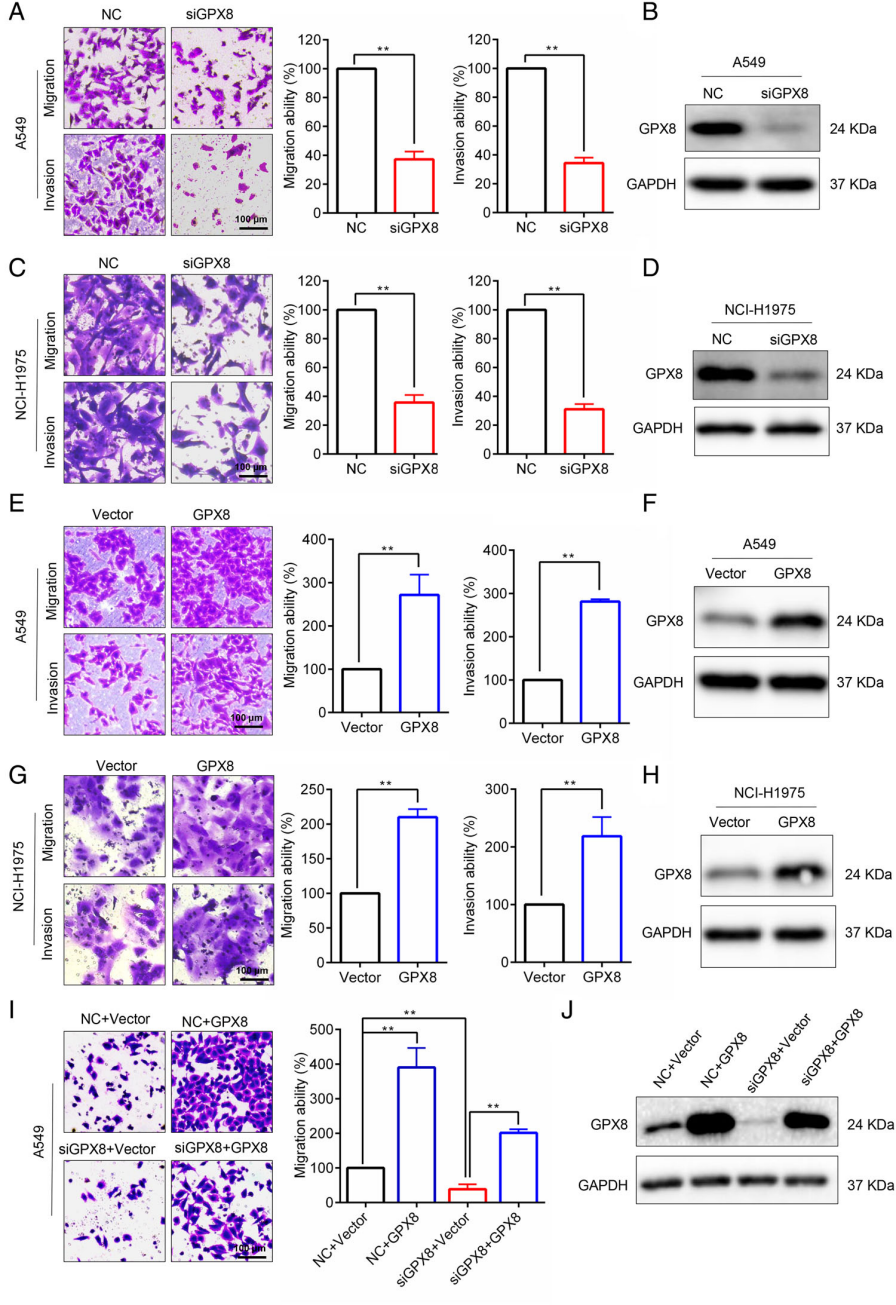
Glutathione peroxidase 8 (GPX8) regulates migration and invasion in lung adenocarcinoma (LUAD) cells. (A) The migration/invasion abilities of A549 cells with downregulation of GPX8 by specific siRNA were conducted by Transwell assay. (B) After knockdown of GPX8 in A549 cells, the protein level of GPX8 was detected using Western blot analysis. (C) The migration/invasion abilities of NCI‐H1975 cells with downregulation of GPX8 by specific siRNA were conducted by Transwell assay. (D) After knockdown of GPX8 in NCI‐H1975 cells, the protein level of GPX8 was detected using Western blot analysis. (E) The migration/invasion abilities of A549 cells with upregulation of GPX8 were conducted by Transwell assay. (F) After overexpression of GPX8 in A549 cells, the protein level of GPX8 was detected using Western blot analysis. (G) The migration/invasion abilities of A549 cells with upregulation of GPX8 were conducted by Transwell assay. (H) After overexpression of GPX8 in A549 cells, the protein level of GPX8 was detected using Western blot analysis. (I) After re‐expression of GPX8 in A549 cell with downregulation of GPX8, the migration ability was performed by Transwell migration assay. (J) The protein level of GPX8 was detected using Western blot analysis. *p < 0.05 and **p < 0.01. Scale bar: 100 µm

To explore the effect of GPX8 in LUAD cell lines, specific siRNA was used to silence GPX8 in several LUAD cells with different molecular backgrounds. Knockdown of GPX8 obviously inhibited migration and invasion abilities in A549 (KRAS mutation), NCI‐H1975 (EGFR mutation; Figure 2A–D). By contrast, overexpression of GPX8 enhanced the abilities of migration and invasion in both A549 cells (Figure 2E,F) and NCI‐H1975 cells (Figure 2G,H). To confirm the on‐target effect of the siRNA, the rescue assay was performed. As shown in Figure 2I,J, re‐expression of GPX8 in A549 cells with downregulation of GPX8 could abolish the migration inhibition by knockdown of GPX8. Therefore, these results indicate that GPX8 regulates migration and invasion in LUAD cells.

### 2.3 Knockdown of glutathione peroxidase 8 inhibits LUAD metastasis in vivo

**FIGURE 3:**
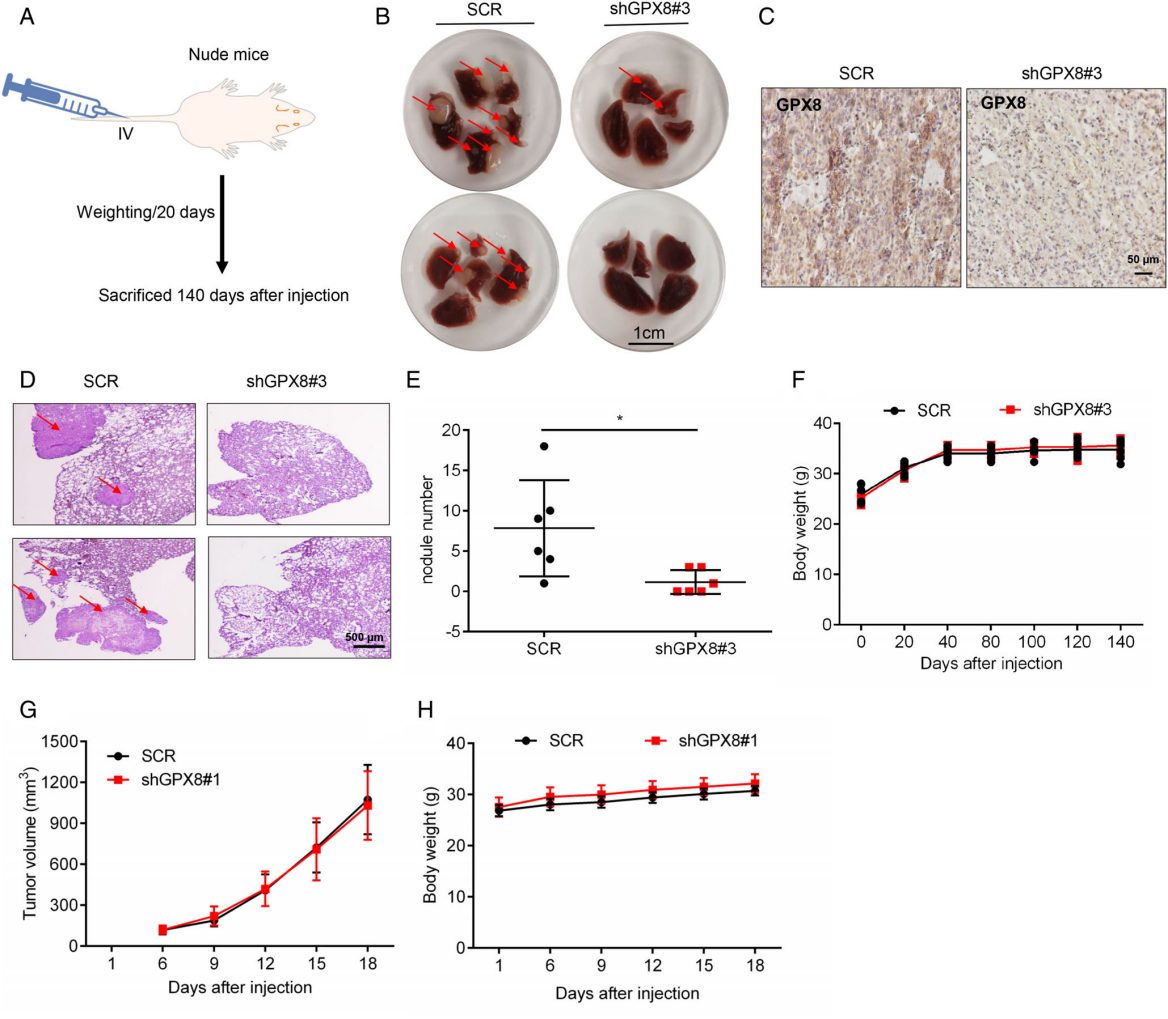
Knockdown of glutathione peroxidase 8 (GPX8) inhibits lung adenocarcinoma (LUAD) metastasis in vivo without tumor growth inhibition in vitro and in vivo. (A) Mice models for GPX8‐mediated metastasis study in vivo. (B) Lung nodule appearance, as indicated by arrows, in nude mice implanted with control (SCR) or GPX8‐downregulation A549 cells (shGPX8#3). Scale bar: 1 cm. (C) GPX8 expression in tumor tissues was detected by immunohistochemical (IHC) staining. Scale bar: 50 µm. (D) H&E staining of metastatic tumors in the lung (*n* = 6). Scale bar: 500 µm. (E) The number of nodules was quantified in the lung. (F) Body weight of nude mice after tail vein injection with the control or GPX8‐downregulation A549 cells. (G) Tumor volume in nude mice (*n* = 8) after subcutaneous injection with the control (SCR) or GPX8‐downregulation NCI‐H1975 cells (shGPX8#1). (H) Body weight of nude mice after subcutaneous injection with the control (SCR) or GPX8‐downregulation NCI‐H1975 cells. *p < 0.05 and **p < 0.01

To further study the role of GPX8 in LUAD metastasis, the stable cell lines with downregulation of GPX8 were established using lenti‐virus system with the specific shRNAs of GPX8 (shGPX8#1 and shGPX8#3). As shown in Figure S2a–S2d, the stable cell lines of A549 and NCI‐H1975 with lower expression level of GPX8 showed much weaker migration and invasion abilities. To evaluate the role of GPX8 in LUAD metastasis in vivo, A549‐SCR and A549‐shGPX8#3 cells were injected into nude mice by the tail vein intravenously. Mice were sacrificed after injection nearly five months (Figure 3A). The metastatic nodules in the lungs were identified. Compared to the lung of mice injected with A549‐SCR cells, only a few metastatic nodules were observed on the lung surface from the mice injected with A549‐shGPX8#3 cells (Figure 3B). As shown in Figure 3C, the expression level of GPX8 detected by IHC staining in the metastatic nodules from SCR group was higher than it from the shGPX8#3 group. The metastatic nodules in the lung were further identified and quantified by HE staining (Figure 3D,E). The body weight of mice in these two groups showed no significant difference (Figure 3F). Next, the function of GPX8 in tumor growth was conducted in vitro and in vivo. Compared to the control groups, cell growth in stable cell lines of A549 and NCI‐H1975 cells with knockdown of GPX8 showed no significant difference (Figure S2E,S2F). Furthermore, the xenograft tumor mouse model was established with injection of NCI‐H1975‐SCR and NCI‐H1975‐shGPX8#1 into the right dorsal flanks of nude mice. The tumor volumes in the two groups showed no obvious difference (Figure 3G) and the body weight of mice in these two groups showed no significant difference (Figure 3H). Collectively, these results show that knockdown of GPX8 inhibits LUAD metastasis without tumor growth inhibition in vitro and in vivo.

### 2.4 Focal adhesion pathway is involved in Glutathione peroxidase 8‐mediated metastasis

**FIGURE 4:**
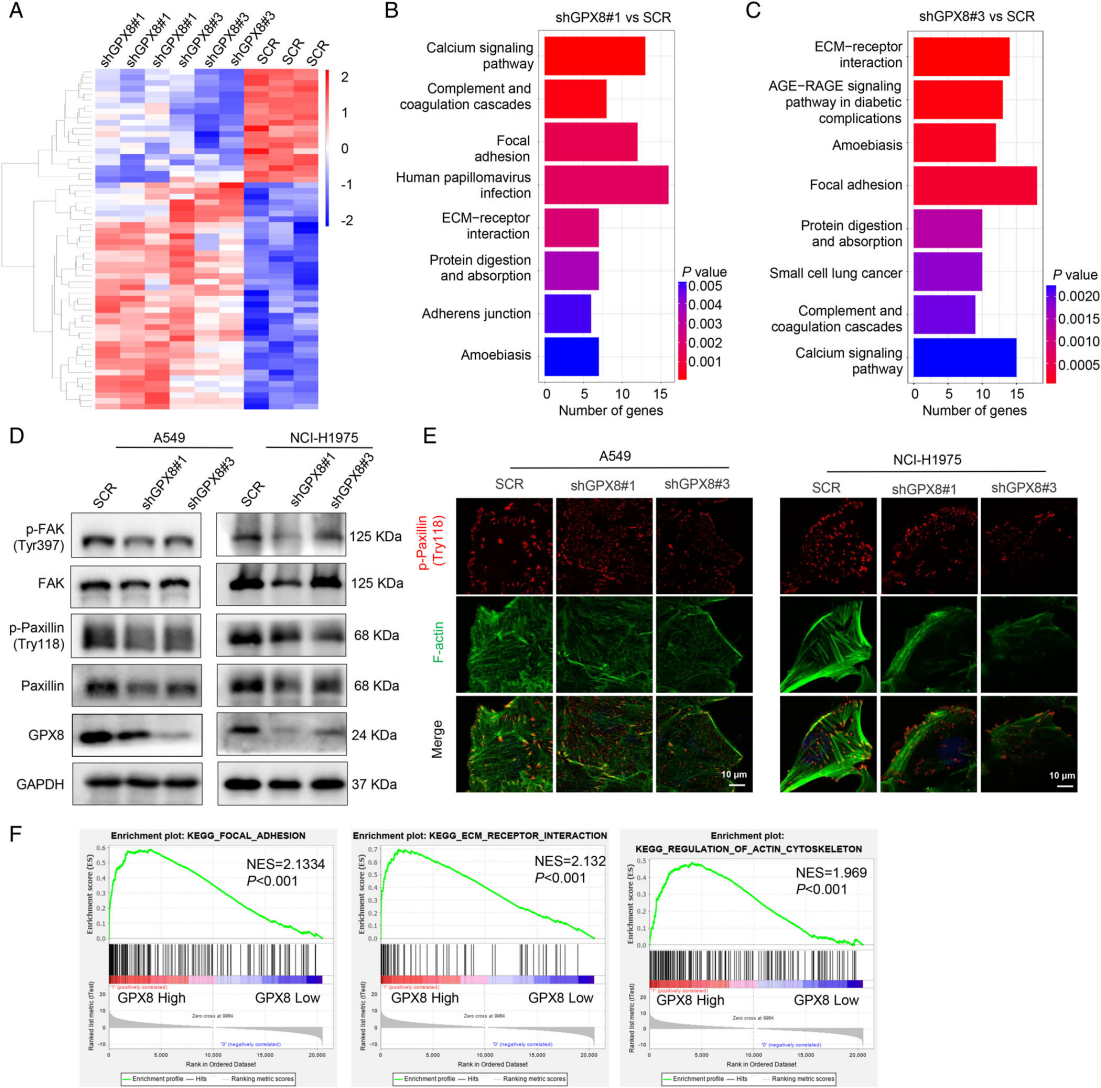
Focal adhesion is involved in Glutathione peroxidase 8 (GPX8)‐regulated metastasis in lung adenocarcinoma (LUAD). (A) The heatmap analysis based on RNA‐seq after knockdown of GPX8 in A549 cells. (B and C) The Kyoto Encyclopedia of Genes and Genomes (KEGG) pathway analysis based on RNA‐seq after knockdown of GPX8. (D) The protein levels of focal adhesion pathway in A549 and NCI‐H1975 cells with downregulation of GPX8 were detected by Western blot analysis. (E) The colocalization of p‐Paxillin and F‐actin in A549 and NCI‐H1975 cells with downregulation of GPX8 was conducted using immunofluorescence. Scale bar: 10 µm. (F) Gene Set Enrichment Analysis (GSEA) analysis of focal adhesion, extracellular matrix (ECM) receptor interaction and regulation of actin cytoskeleton signatures between GPX8 high and low group in TCGA_LUAD dataset

To explore the mechanism of GPX8‐mediated metastasis in LUAD, the RNA sequencing (RNA‐seq) was performed in A549‐SCR, A549‐shGPX8#1, and A549‐shGPX8#3 cells. The heatmap showed the differential expressed genes after knockdown of GPX8 (Figure 4A) and the Kyoto Encyclopedia of Genes and Genomes (KEGG) enrichment analysis indicated the potentially GPX8‐regulated pathways based on RNA‐seq (Figure 4B,C). The focal adhesion pathway and extracellular matrix (ECM) receptor interaction, which are closely associated with metastasis, were enriched in both two clones A549‐shGPX8#1 (Figure 4B) and A549‐shGPX8#3 (Figure 4C), compared to A549‐SCR. FAK and Paxillin localized to focal adhesion sites are two important proteins in focal adhesion turnover and transmitting signals downstream of integrins.^33^, ^34^ As shown in Figure 4D, the expression levels of p‐Paxillin (Tyr118) and p‐FAK (Try397) were obviously decreased in A549 and NCI‐H1975 cells after downregulation of GPX8. Paxillin is an adaptor protein colocalized with actin filaments and signaling proteins in cell motility.^35^ The colocalization of p‐Paxillin and F‐actin in A549 and NCI‐H1975 cells was conducted using immunofluorescence, which showed knockdown of GPX8 impaired the actin polymerization and colocalization of p‐Paxillin with actin filaments (Figure 4E). After knockdown of GPX8, the p‐Paxillin expression (Tyr118) was also downregulated in vivo by IHC staining from the mice by tail vein injection (data not shown). Furthermore, the focal adhesion, ECM receptor interaction, and regulation of actin cytoskeleton pathways were enriched by GSEA analysis in the samples with high expression of GPX8 (the upper quartile) in TCGA_LUAD dataset (Figure 4F), which provided more evidence for the mechanism in GPX8‐mediated metastasis in LUAD. Based on these findings, focal adhesion‐associated proteins, Paxillin and FAK, are involved in GPX8‐regulated metastasis in LUAD.

### 2.5 Glutathione peroxidase 8 is involved in cancer‐associated fibroblasts ‐mediated migration promotion of lung cancer cells

**FIGURE 5:**
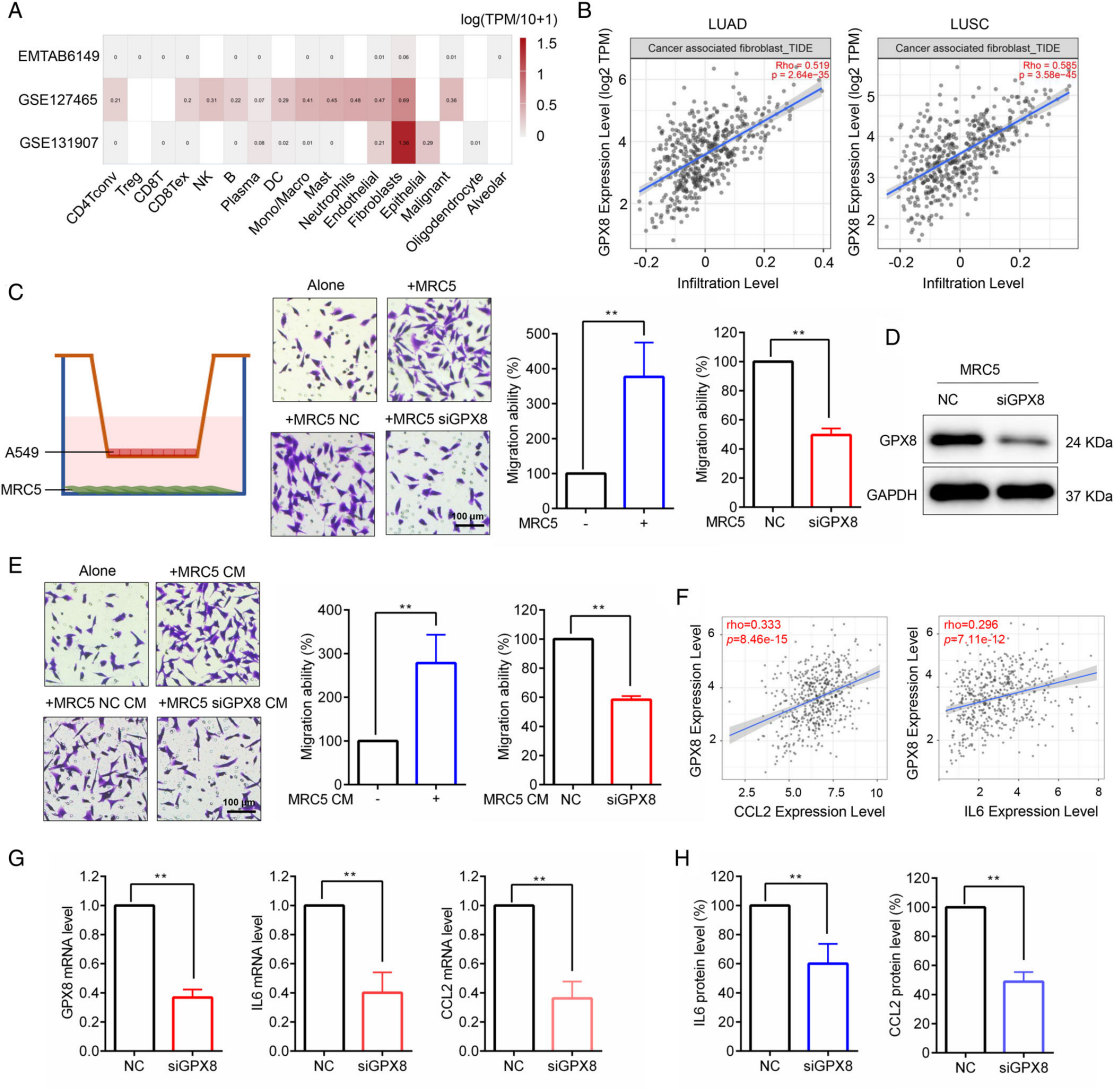
Glutathione peroxidase 8 (GPX8) was involved in CAFs‐mediated migration promotion of lung cancer cells. (A) Expression level of GPX8 in different cells of lung cancer tumor microenvironment (TME) in TISCH database. (B) Correlations between GPX8 expression and CAF infiltration in lung adenocarcinoma (LUAD) and LUSC patients. (C) In the coculture system, the migration ability of A549 cells was performed by using Transwell migration assay. (D) The protein level of GPX8 in MRC5 cells was detected by Western blot analysis. (E) The migration ability of A549 cells in conditioned media (CM) of indicated MRC5 cells was conducted through Transwell migration assay. (F) Correlation analysis between GPX8 expression and IL6/CCL2 in lung cancer based on TIMER2.0 database. (G) After knockdown of GPX8 in MRC5 cell, the mRNA expression level of GPX8, CCL2, and IL6 were tested by qPCR. (H) After knockdown of GPX8 in MRC5 cell of the coculture system, the secretion of IL6 and CCL2 were detected using ELISA assay. *p < 0.05 and **p < 0.01. Scale bar: 100 µm

TME is important for lung cancer progression and metastasis. Single‐cell RNA sequencing (scRNA‐seq) provides gene expression profile of individual cells in TME. As shown in Figure 5A, the expression level of GPX8 was much higher in fibroblasts than in other cell types based on scRNA‐seq data analysis (EMTAB6149,^36^ GSE127465,^37^ and GSE131907^38^) of lung cancer from the Tumor Immune Single‐cell Hub (TISCH) database. The GPX8 expression level was positively associated with CAF infiltration in lung cancer (Figure 5B). To further explore the potential role of GPX8 in CAF, the coculture system of A549 cells and MRC5 cells was established. As shown in Figure 5C, MRC5 obviously facilitated A549 cell migration, but GPX8 silence (Figure 5D) in MRC5 cells reversed the migration promotion of A549 cells in the coculture system. Furthermore, conditioned media from MRC5 promoted A549 cell migration and conditioned media from MRC5 with downregulation of GPX8 suppressed A549 cell migration (Figure 5E). Interleukin (IL6) and C‐C motif chemokine ligand 2 (CCL2), two cytokines secreted by CAF, play important role in cancer cell migration. As shown in Figure 5F, the expression level of GPX8 was positively correlated with CCL2 and IL6 in lung cancer. Moreover, knockdown of GPX8 in MRC5 cells decreased mRNA expression (Figure 5G) and secretion (Figure 5H) of CCL2 and IL6 in the coculture system. These findings suggested that high expression of GPX8 in CAF promoted lung cancer cell migration possibly through secretion of CCL2 and IL6.

### 2.6 Bromodomain and extra‐terminal inhibitor modulates glutathione peroxidase 8 expression and lung cancer cell migration

**FIGURE 6:**
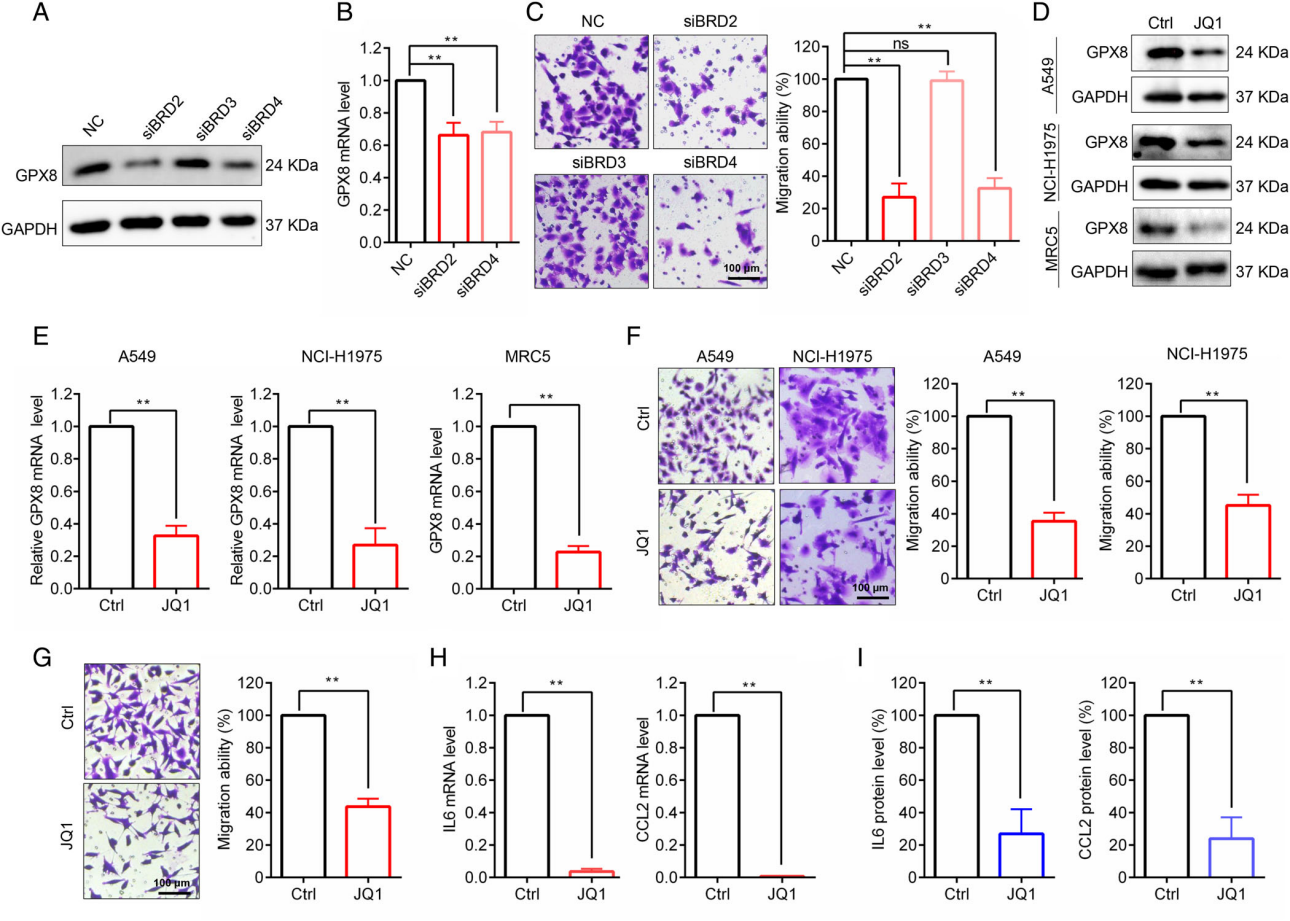
Bromodomain and extra‐terminal (BET) protein regulated Glutathione peroxidase 8 (GPX8) expression and lung cancer cell migration. (A) The protein level of GPX8 in A549 cell with knockdown of BRD2, BRD3, and BRD4 was detected by Western blot analysis. (B) The mRNA level of GPX8 in A549 cell with knockdown of BRD2 and BRD4 was detected by qPCR. (C) The migration ability of A549 cells with knockdown of BRD2, BRD3, and BRD4 was detected by Transwell assay. (D) The protein level of GPX8 was detected by Western blot analysis in A549, NCI‐H1975, and MRC5 cells after treatment with 1 µM BET inhibitor JQ1 for 24 h. (E) The mRNA level of GPX8 was tested by qPCR in A549, NCI‐H1975, and MRC5 cells treated with JQ1. (F) After treatment with JQ1, the migration ability of A549 and NCI‐H1975 cells was detected by Transwell assay. (G) In the coculture system, the migration ability of A549 cells was conducted by Transwell assay after treatment with JQ1. (H) The mRNA level of IL6 and CCL2 in MRC5 cells treated with JQ1 was tested by using qPCR. (I) After treatment with JQ1 in the coculture system, the secretion of IL6 and CCL2 were detected using ELISA assay. *p < 0.05 and **p < 0.01. Scale bar: 100 µm

The results indicated that GPX8 was obviously overexpressed in the LUAD patients both in the mRNA and protein levels (Figure 1E–G), suggesting that transcriptional activation of GPX8 mainly contributed to the increased protein expression of GPX8 in LUAD metastasis. Thus, the upstream regulatory of GPX8 at the transcriptional level was further explored. Based on ChIP‐seq data analysis by using Toolkit for Cistrome Data Browser (http://dbtoolkit.cistrome.org/), several transcription regulators that had the regulatory potential on GPX8 were identified (Figure S4). Of note, the BET protein BRD2 and BRD4 were selected for further analysis, as it has relatively higher regulatory potential score and it is druggable by BET inhibitors which are under evaluation in clinical trials.^39^ Genetic silence of BRD2 and BRD4 with specific siRNAs could reduce the protein (Figure 6A) and the transcription levels of GPX8 (Figure 6B) in A549 cells. Knockdown of BRD2 and BRD4 obviously inhibited migration of A549 cells (Figure 6C). As expected, the BET inhibitor JQ1 could downregulate GPX8 expression in lung cancer A549 and NCI‐H1975 cells, and lung fibroblast MRC5 cells both in the protein level (Figure 6D) and mRNA level (Figure 6E). After treatment with JQ1 for 24 h, the migration ability was decreased in A549 and NCI‐H1975 cells (Figure 6F), while JQ1 showed no significant proliferation inhibition within 24 h treatment time (data not shown). In the coculture system, JQ1 also inhibited A549 cell migration (Figure 6G). Furthermore, after treatment with JQ1, the mRNA level of CCL2 and IL6 was decreased in MRC5 cells (Figure 6H) and the secretion of CCL2 and IL6 was inhibited in the coculture system (Figure 6I). Thus, BRD2 and BRD4 could regulate GPX8 expression and BET inhibitors may serve as a treatment strategy for GPX8‐driven lung cancer metastasis.

## 3 DISCUSSION

Metastasis is the spread of cancer from its primary site to other parts of the body.^8^ It is quite common for LUAD to metastasize before it is diagnosed in the clinic, which is mainly responsible for the great majority of LUAD deaths.^4^ LUAD metastasis is a multi‐step process with a variety of regulatory mechanisms.^40^ Many mechanisms and molecules have been investigated in LUAD metastasis, such as the genes picked out together with GPX8 in this study, but there are still no targetable molecules used for metastatic LUAD in clinic and the treatment strategy for metastatic LUAD is still not effective. In the current study, GPX8 was identified as novel pro‐metastatic factor in lung cancer based on datasets analysis and experimental investigation. GPX8 expression was positively correlated with other metastasis promoting genes (Figure S1), indicating the pro‐metastatic function of GPX8 in LUAD. In fact, high expression of GPX8 is negatively associated with the survival time of the patients with gastric cancer^19^ and breast cancer,^20^ suggesting its important role in the cancer development. Moreover, GPX8 was highly expressed in the tumor tissues compared to the normal tissues (Figure 1E–G, J), indicating that targeting GPX8 may provide therapeutic benefit with little side effect. Metastasis is constantly occurred in the LUAD patients with late stage.^41^ The patients in the late stage had higher expression levels of GPX8 compared to early stage (Figure 1H), further supporting the metastatic role of GPX8 in LUAD. As expected, our findings pointed out that GPX8 facilitated the metastatic phenotype in LUAD in vitro and in vivo. Many oncogenic driver mutations are identified in LUAD and EGFR/KRAS are the predominant driver gene mutations accounting nearly 50%.^42^, ^43^, ^44^ The mRNA level of GPX8 was not significantly changed between different molecular subtypes of LUAD (Figure S1H) and knockdown of GPX8 inhibited migration and invasion in A549 cells with KRAS mutation (Figure 2A), NCI‐H1975 cells with EGFR mutation (Figure 2C), and NCI‐H1299 cells with NRAS mutation (data not shown), suggesting that GPX8 promotes metastasis independent of molecular subtypes in LUAD. These findings indicate that GPX8 is a novel biomarker and promising therapeutic target for metastatic LUAD.

To further study the mechanism of GPX8‐mediated metastasis in LUAD, RNA‐seq analysis in LUAD cells with downregulation of GPX8 was performed. Focal adhesion and ECM receptor interaction were enriched by KEGG analysis. Focal adhesion is a large complex containing FAK, Paxillin, talin, vinculin, etc., which links the ECM to the actin cytoskeleton through the ECM receptor integrins and mediates signal transduction initiating signaling pathways in response to adhesion.^45^ To our result, knockdown of GPX8 obviously downregulated expression of p‐FAK and p‐Paxillin. FAK and Paxillin are two important components of focal adhesion and are closely linked to cancer metastasis.^46^, ^47^ Paxillin is phosphorylated by active FAK to recruitment of other components of focal adhesion and transduction of numerous signaling cascades in promoting cell motility.^33^ It was also identified in the result that suppression of GPX8 reduced the colocalization of Paxillin with F‐actin, thereby leading to the blocking of signal transduction between ECM and actin cytoskeleton. Moreover, the role of focal adhesion, ECM receptor interaction, and regulation of actin cytoskeleton in GPX8‐mediated LUAD metastasis were further confirmed by GSEA analysis in the LUAD patients with high expression of GPX8.

However, GPX8 is located in ER membrane, which is difficult to regulate focal adhesion directly. Calcium signal pathway was also enriched based on the RNA‐seq. Ca^2+^ is a critical regulator for cell migration by regulation of focal adhesion turnover.^48^ GPX8 is essential for regulating Ca^2+^ dynamics in cytosol, ER, and mitochondria in Hela cells.^17^ Thus, Ca^2+^ may be the regulator between GPX8 and focal adhesion, although the Ca^2+^ in cytosol had no significant difference between the A549 cells with different expression level of GPX8 (data not shown), which was possibly due to the low signal of Ca^2+^ in cytosol without any external stimulation. It may need to further study the calcium signal alteration when cells are in motion and uncover the role of Ca^2+^ in GPX8‐regulated metastasis in LUAD. Furthermore, GPX8 has been identified as a mesenchymal metabolic signature gene in EMT transition.^49^ Recently, it was reported that lack of GPX8 suppresses the aggressive phenotype and stemness features in breast cancer cells.^20^ EMT and stemness could enhance metastatic phenotype in cancer progress.^50^, ^51^ However, knockdown of GPX8 did not change cell morphology and the expression of stemness markers (CD44 and CD133) obviously in LUAD cells (data not shown). These results suggested that the detailed role of GPX8 differs from cancer types and needs to be further studied.

TME plays an important role in cancer progression and metastasis, of which CAF is a predominant stromal cell type with significant heterogeneity and plasticity.^52^ CAFs serve as essential regulator at all stages of cancer progression, including cancer metastasis, which is a potential strategy for cancer treatment.^52^ Our finding indicated that GPX8 was highly expressed in CAF and closely associated with CAF infiltration in lung cancer (Figure 5A,B). CAF infiltration contributes to cancer metastasis through secretion of cytokines, interaction with tumor cells, regulation of immune response, and ECM remodeling.^52^ In the coculture system, knockdown of GPX8 influenced CCL2 and IL6 expression and secretion. CCL2, a crucial chemokine in TME, functions mainly through binding to its receptor CCR2 and recruit macrophage and monocyte, which promotes cancer progression and metastasis.^53^ IL‐6 is a key cytokine secreted from CAF, which will induce immunosuppression through inhibition of T cell response and regulation of metabolism.^54^, ^55^ CAF‐produced IL6 also could promote cancer metastasis through TGFβ‐induced EMT and STAT3 signal.^56^ Intriguingly, it was reported that GPX8/IL6/STAT3 axis was important for maintaining aggressive phenotype in breast cancer.^20^ Furthermore, the result showed that the expression level of GPX8 was positively correlated with IL6 and CCL2 in lung cancer. Thus, GPX8‐mediated secretion of IL6 and CCL2 by CAF in TME may promote lung cancer metastasis.

GPX8, which shows pro‐metastatic role both in cancer cells and CAFs, is a distinguishably promising target for lung cancer metastasis, so it is meaningful to explore potential strategies against GPX8‐mediated metastasis. The enzyme activity of the GPX family with a highly conserved catalytic center could maintain redox homeostasis. Although GPX8 peroxidase could prevent leakage of H_2_O_2_ from ER, compared with other GPX members, GPX8 has too weak activity in ROS clearance.^16^, ^57^ GPX8 regulated Ca^2+^ dynamics was independent of its enzyme activity,^17^ indicating that GPX8 has other functions in addition to the enzyme activity. As shown in Figure S3A, knockdown of GPX8 did not induce obvious ROS generation in LUAD cells. The enzyme inactive mutation (C79S) plasmid of GPX8 fusion with FLAG was constructed (Figure S3B) and transfected into A549 cells colocalized with Calnexin, an ER membrane marker (Figure S3C, D). GPX8‐Mut showed a similar effect on cell migration promotion as the WT form (Figure S3E), indicating that GPX8‐regulated metastasis was possibly independent of its enzyme activity. Thus, downregulation of GPX8 is a better approach to suppress its pro‐metastatic function in lung cancer. In this study, pharmacologic inhibition and genetic silence of BRD2/BRD4 obviously downregulated GPX8 expression and prevented migration both in lung cancer cells and CAF. BRD2/BRD4 are the potential regulator for GPX8 expression, but the detailed mechanism of BET proteins involved in transcriptional regulation of GPX8 in this study has not been clearly verified, which still needs to be further explored in the future. BET inhibitors are promising epigenetic drugs under clinical estimation in various cancers, including lung cancer,^39^, ^58^ which serves as an alternative strategy against GPX8‐facilitated metastasis. Besides, targeting protein degradation has emerged as a strategy in cancer therapy via PROTAC or deubiquitylase enzyme inhibitors,^59^, ^60^ which may also be a potential strategy of targeting GPX8 for LUAD metastasis with the further study of GPX8 degradation in the future.

## 4 CONCLUSIONS

**FIGURE 7:**
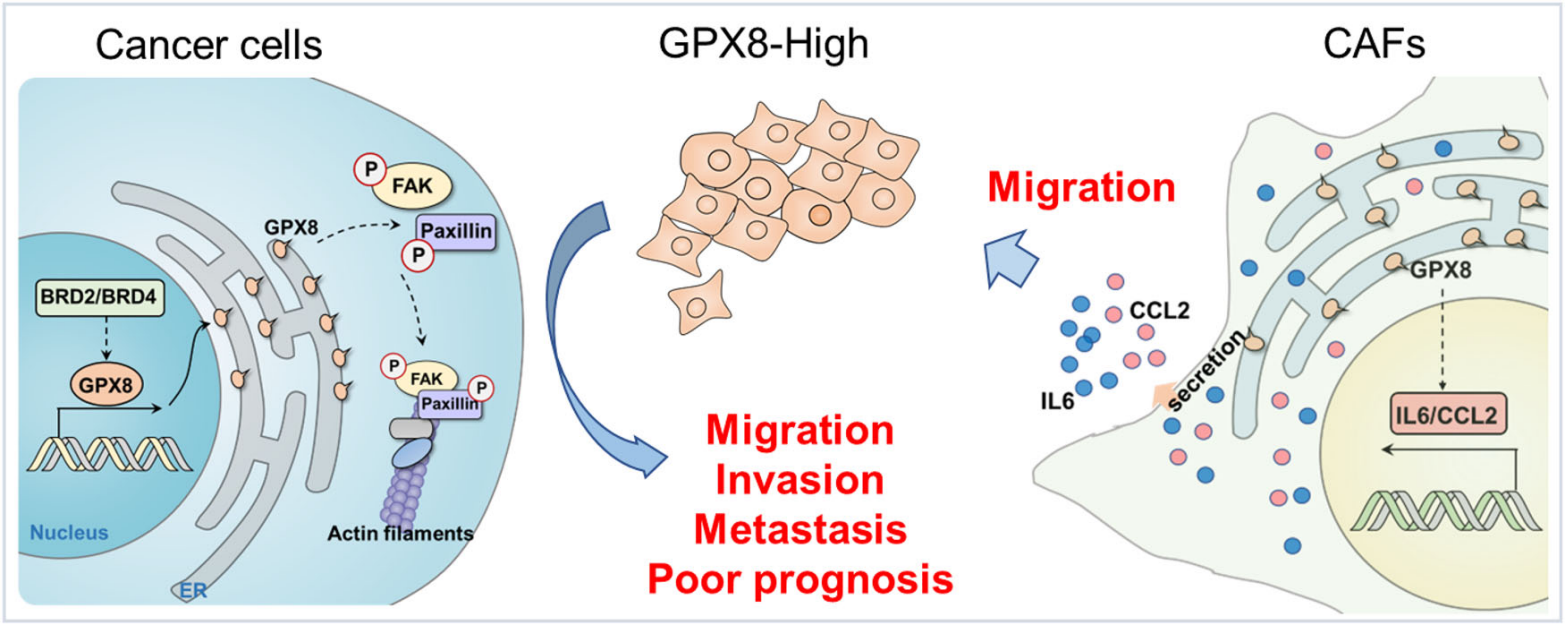
High expression of Glutathione peroxidase 8 (GPX8) on cancer cells and CAFs promote lung cancer metastasis. BRD2/BRD4‐mediated upregulation of GPX8 promotes lung cancer metastasis by the regulation of p‐FAK and p‐Paxillin. GPX8‐mediated IL6 and CCL2 production of CAF may play crucial role for lung cancer metastasis

In summary (Figure 7), GPX8 serves as a pro‐metastatic factor both in tumors and TME, which is a potentially therapeutic target in lung cancer. Knockdown of GPX8 obviously inhibited LUAD metastasis through the modulation of focal adhesion pathway and GPX8‐mediated IL6 and CCL2 production of CAF may play crucial role for lung cancer metastasis. BET proteins are involved in transcriptional regulation of GPX8 and BET inhibitor is a potential strategy against GPX8‐mediated lung cancer metastasis.

## 5 MATERIALS AND METHODS

### 5.1 Antibodies and chemicals

The primary antibody of GPX8 (16846‐1‐AP) was acquired from Proteintech Group (Rosemont, IL, USA). The primary antibodies against p‐FAK (Tyr397, #3283), FAK (#71433), p‐Paxillin (Tyr118, #2541), Paxillin (#2542), and GAPDH (#5174) were obtained from Cell Signaling Technology (Beverly, MA, USA). The secondary HRP‐linked antibody (anti‐rabbit IgG, #7074) was purchased from Cell Signaling Technology. The Alexa Fluor® 594 conjugated secondary antibody (#A‐11001) was purchased from Thermo Fisher Scientific (Waltham, MA, USA). Actin‐Tracker Green, Hematoxylin and Eosin Staining Kit, and Crystal violet were acquired from Beyotime Biotechnology Corporation (Shanghai, China). JQ1(S7110) and PFI‐1(S1216) were purchased from Selleck Chemicals (Houston, TX, USA).

### 5.2 Cell lines and cell culture conditions

The cell lines A549 and NCI‐H1975 were obtained from American Type Culture Collection (Rockville, MD, USA) and grown in RPMI‐1640 medium supplemented with 10% fetal bovine serum and 1% penicillin/streptomycin (Gibco, Carlsbad, CA, USA). HEK‐293T was a generous gift from Prof. Chung Hang LEUNG (University of Macau). HEK‐293T cells were grown in Dulbecco's Modified Eagle Medium containing 10% fetal bovine serum. The lung fibroblast cell line MRC‐5, grown in Minimum Essential Medium supplemented with 10% fetal bovine serum and 1% penicillin/streptomycin, was obtained from Shanghai Cell Bank (Shanghai, China). The cells were cultured in a humidified incubator containing 5% CO_2_ at 37°C. The short tandem repeat (STR) DNA profiling analysis was used for human lung cancer cell line authentication (Shanghai Genesky Biotechnologies Inc.) on July 19, 2019.

### 5.3 Transwell migration and invasion assays

The abilities of cell migration and invasion were measured by using Transwell assay. For the migration assay, the RPMI‐1640 medium containing 10% fetal bovine serum was added into the lower chambers, then the cells suspended in the medium without serum at a density of 3 × 10^4^ cells/well were seeded into the upper chambers of the Transwell inserts. For the invasion assay, the upper chambers were freshly coated with Matrigel (Corning Life Science, Tewksbury, MA, USA) at 37°C for 1 h before cells seeding. After 24 h incubation, the cells on the upper surface of the Transwell insert were removed by cotton swabs. For the coculture system, the MRC‐5 cells were seeded into the 24‐well plate in the complete medium and A549 cells suspended in the FBS free medium were seeded into the Transwell inserts in upper chambers. After 16 h incubation, the cells on the upper surface of the Transwell insert were removed by cotton swabs. Subsequently, the migratory or invasive cells on the bottom surface of the Transwell insert were fixed with 4% PFA and stained with crystal violet staining solution. The staining cells in three random fields were photographed and quantified by using the Olympus IX73 Inverted Microscope (Tokyo, Japan).

### 5.4 siRNA interference

The cell suspensions were seeded into six well‐plates with a density of 1.5 × 10^5^ cells/well. The cells were cultured overnight and transfected with specific siRNA according to manufacturer's instruction of Lipofectamine 2000 transfection reagent (Invitrogen Corp., Carlsbad, CA, USA). After 6 h incubation, the cells were replaced with fresh culture medium and grown overnight for the following work. The siRNAs were obtained from GenePharma (Shanghai, China) and the sequences were presented in Table S1.

### 5.5 Western blot analysis

Western blot analysis was conducted as our previous study.^61^ Briefly, cells were washed by precooling PBS and lysed by using RIPA buffer containing phosphatase inhibitor cocktail and protease inhibitor PMSF. The concentrations of the total proteins were detected by using Pierce™ bicinchoninic acid (BCA) protein assay kit (Thermo Fisher Scientific, Waltham, MA, USA). The same amounts of protein were loaded into wells and separated in gradient sodium dodecyl sulfate polyacrylamide (SDS‐PAGE)gels. The proteins on the gels were transferred to polyvinylidene fluoride (PVDF) membranes. The membrane blocking was performed with 5% nonfat dry milk for 1 h at room temperature. After incubation with specific primary antibodies at 4°C overnight, the membranes were washed with phosphate buffered solution with Tween‐20 (PBST) and incubated with the horseradish peroxidase (HRP)‐conjugated secondary antibodies at room temperature for 1 h. The immunoreactive signals were visualized with ECL Western blot detection reagent (GE Healthcare, Chicago, IL, USA).

### 5.6 Plasmid construction and transfection

For GPX8 overexpression, the primers were designed with the restriction sites of EcoR I and Not I, and used to amplify the coding sequence (CDS) of human GPX8. The CDS of GPX8 was cloned into pCDH‐puro plasmid to construct the GPX8 overexpression plasmid. The cell suspensions (1.5 × 10^5^ cells/well) were seeded into six well‐plates and cultured overnight. The cells were transfected with the indicated plasmids by using TurboFect transfection reagent (Invitrogen Corp., Carlsbad, CA, USA) for 48 h and collected for the following work.

### 5.7 Establishment of stable cell lines with lentiviral infections

To establish stable cells in A549 and NCI‐H1975 cells with downregulation of GPX8, the human GPX8‐specific small hairpin RNAs (shRNA) were cloned into pLKO.1puro plasmids. The sequences of GPX8 shRNAs were designed online by using the RNAi Consortium tool (http://www.broadinstitute.org/rnai/trc) and synthesized by Invitrogen Life Technologies (Shanghai, China). The plasmids mixed with the recombinant pLKO.1puro, pCMV‐dR8.2 dvpr, and pCMV‐VSVG were transfected together into 293T cells. After incubation for 60 h, the collected supernatant was filtered by using 0.45 µm‐diameter filters. Then, the A549 and NCI‐H1975 cells were infected with the supernatant and replaced with fresh culture medium after 24 h infection. Subsequently, the cells with downregulation of GPX8 were selected in the presence of puromycin for 5 days. The efficacy of GPX8 shRNAs was verified by the Western blot analysis. The sequences of GPX8 shRNAs and scramble (SCR) were presented in Table S1.

### 5.8 RNA sequencing and Kyoto Encyclopedia of Genes and Genomes analysis

RNA‐seq was conducted by Omigen (Hangzhou, China) as described in our previous study.^62^ Briefly, the RNA‐seq reads were mapped to the UCSC Genome Browser (GRCh37/hg19) reference using STAR version 2.4.1d. The gene expression levels were quantified by using Partek E/M annotation model normalized to the total counts (Table S2). The differentially expressed genes were analyzed using Partek Genomics Suite. Genes showing significantly (*p* < 0.05 and more than 1.5‐fold changes) differentially expressed were further used to perform KEGG pathway analysis by using the online DAVID 6.8 bioinformatics resources.

### 5.9 RNA preparation and qPCR

The TRIzol® Reagent (Life Technologies, Shanghai, China) was used to extract the total RNA of indicated cells. The concentrations of total RNA were detected by NanoDrop spectrophotometers (Thermo Fisher Scientific, Waltham, MA, USA). Then, the same amounts of total RNA were used to synthesize the first‐strand cDNA using the RevertAid First Strand cDNA Synthesis Kit (Thermo Fisher Scientific, Waltham, MA, USA). The cDNA was applied for quantitative real‐time polymerase chain reaction (qPCR) by using the FastStart Universal SYBR Green Master kit (Roche, Shanghai, China) on Mx3005P real‐time PCR system (Agilent Technologies, Santa Clara, CA, USA). GAPDH was used for normalization with target genes. The qPCR primers were shown in Table S1.

### 5.10 Immunofluorescence

The stable cell lines of A549 and NCI‐H1975 with knockdown of GPX8 were seeded into confocal dishes (SPL Life sciences, Pocheon, Korea). After incubation for 48 h, the cells were washed using PBS and fixed with 4% PFA at room temperature for 30 min. After permeabilization with 0.5% Triton X‐100, the cells were hydrated with PBS for 2 h and blocked using 5% BSA for 1 h at room temperature. Afterward, the cells were sequentially incubated with the indicated primary antibodies at 4°C overnight and the Alexa Fluor® 594‐conjugated antibody for 1 h at room temperature. Actin‐Tracker Green was used to stain the F‐actin and Hoechst 33342 was used to highlight the nuclei in the cells. After a wash step in PBS, the cells were captured by using a Leica TCS SP8 confocal laser scanning microscope (Solms, Germany).

### 5.11 Animal studies

The animal model of LUAD metastasis in nude mice was established by tail vein injection. Five‐week‐old male nude mice (BALB/C) provided by University of Macau were used for LUAD metastasis study and all the experiments were approved by the Animal Research Ethics Committee of the University of Macau (UMARE‐022‐2019). The cells (A549‐SCR and A549‐shGPX8#3) (5 × 10^6^ cells/mouse) were trypsinized, resuspended, washed with PBS, and injected into the mice by tail vein injection. Each group contained six mice. The weight of mice was monitored using electronic scale every 20 days. After nearly five months, the mice were sacrificed and dissected. The lungs were photographed and the metastatic nodules on the surface of the lung were counted. The lung tissues were fixed in 4% PFA for 24 h and embedded with paraffin for following detection work.

The subcutaneous tumor mice models were also conducted to verify the role of GPX8 in tumor growth. The NCI‐H1975‐SCR and NCI‐H1975‐shGPX8#1 cells (1.5 × 10^6^ cells/mouse) were injected subcutaneously into the right armpit of each mouse. Each group contained eight mice. The size of tumors was measured by using vernier calipers every 3 days and the tumor volume was calculated according to the formula: volume = length × width^2^/2. The weight of mice was also monitored and recorded every 3 days. After injection for 18 days, the mice were sacrificed.

### 5.12 Hematoxylin eosin staining assay

The hematoxylin eosin (HE) stain was an approach widely used in histology and a common method for medical diagnosis. In our study, the HE staining assay was used to identify the metastatic nodules in lungs from mice models. The paraffin‐embedded tissues were cut into sections with 4 µm and mounted onto microscope slides by using Microtomes (Leica Biosystem, Buffalo Grove, IL, USA). The sections were deparaffinized in xylene, followed by rehydration in graded concentrations of ethanol. The sections were stained with haematoxylin and eosin, then dehydrated in graded ethanol and xylene. Subsequently, the neutral gum was used to cover the slides together with coverslip slides. The metastases in lung tissue sections were observed and photographed by using Nikon ECLIPSE Ci Upright Clinical Microscope (Melville, NY, USA).

### 5.13 Immunohistochemical analysis

The protein level of GPX8 on the human LUAD paraffin embedded tissue array (HLugA150CS02, Outdo Biotech, Shanghai, China) with 75 paired cases (tumor and normal adjacent tissues) and the metastatic nodules from mice models were performed by IHC analysis. After dewaxing in xylene, the section was rehydrated with graded concentrations of ethanol. For antigen unmasking, the section was treated with a microwave oven for 15 min in citrate buffer solution (pH 6.1). Then, to block the endogenous peroxidase activity, the section was incubated in Avidin/Biotin Blocking Kit provided by Vector Laboratories (Burlingame, CA, USA). Subsequently, the section was incubated with primary anti‐GPX8 (Abcam, Cambridge, UK) in 4°C overnight. After washing with PBS, the section was incubated sequentially with anti‐rabbit biotin‐labeled secondary antibodies, followed by incubation of streptavidin‐conjugated peroxidase. The protein signal was visualized using the DAB IHC Detection Kit provided by Vector Laboratories (Burlingame, CA, USA). The pictures were photographed by using Nikon ECLIPSE Ci Upright Clinical Microscope (Melville, NY, USA). The IHC scores were based on percentage scores and the intensity scores. The percentage of staining was scored (*P*) as 0, 1, 2, 3, and 4 for no positive cells, < 25% of positive cells, 25%–50% of positive cells, 50%–75% of positive cells, and > 75% of positive cells, respectively. The intensity score (*I*) of GPX8 staining was ranked as 0 (no staining), 1 (weak), 2 (moderate), and 3 (strong). The IHC score of GPX8 staining in each sample was calculated according to the formula: IHC score = *P* × *I*. The samples were classified according the IHC score as negative (final score = 0), low (0 < final score < 6), and high (final score ≥ 6).

### 5.14 IL6 and CCL2 ELISA

The CCL2 and IL6 secretion in the coculture system was detected by using ELISA kits (Human CCL2/MCP‐1 ELISA Kit, PC130; Human IL‐6 ELISA Kit, PI330) bought from Beyotime Biotechnology Corporation (Shanghai, China). After centrifugation, the supernatant in the coculture system was collected for ELISA detection according to manufacturer's instruction. The concentration of CCL2 and IL6 was calculated based on the standard curve line.

### 5.15 Data analysis

LUAD metastasis genes were analyzed by comparing murine lung primary adenocarcinomas and their metastases from two Gene Expression Omnibus (GEO) datasets (GSE84447 and GSE40222).^9^, ^63^ Genes with alteration of *p* < 0.05 and more than two‐fold changes were considered differentially expressed. The GEPIA database (http://gepia2.cancer‐pku.cn/) was used to perform survival analysis of the metastasis genes by using TCGA_LUAD dataset. The mRNA levels of GPX8, NKX2‐1, and HMGA2 in TCGA_LUAD dataset were downloaded from R2: Genomics Analysis and Visualization Platform (http://r2.amc.nl). The correlations between GPX8 and other genes were analyzed by using Pearson correlation coefficient. The protein levels of GPX8 in LUAD normal and tumor tissues were derived from Xu2020_LUAD cohort with 83 paired LUAD tissues and normal lung tissues^64^ and Gillette2020_LUAD cohort with 101 paired LUAD tissues and normal lung tissues.^65^ The difference was analyzed using unpaired Student's *t* test. TCGA_LUAD (RNA Seq V2 RSEM) data with 515 LUAD tissues and 59 normal lung tissues were derived from the cBioPortal website (https://www.cbioportal.org/). Samples with GPX8 mRNA level below the lower quartile represented low group, and those above the upper quartile represented high group. Gene Set Enrichment Analysis (GSEA) version v4.1.0 was used to enrich the canonical pathway in c2.cp.kegg.v7.1.symbols between the high group and the low group. GPX8 mRNA and protein levels for survival analysis were obtained from TCGA_LUAD dataset and Xu2020_LUAD cohort, respectively. GPX8 expression below the lower quartile was considered to have a low expression, and that above the upper quartile was considered to have a high expression. Kaplan–Meier (KM) survival curves were generated for the survival analyses. Statistical significance was conducted by using the log‐rank test.

### 5.16 Statistical analysis

The significance between two groups was conducted by using unpaired Student's *t* test. KM plot and the log‐rank test were utilized to do survival analysis. The correlation analyses between two groups were performed with Pearson correlation coefficients. The data analyses were analyzed by using GraphPad Prism software 6 (GraphPad Software, Inc., La Jolla, CA, USA). The data were represented as mean ± SD. A *p*‐value < 0.05 was considered as a significant change.

## CONFLICT OF INTEREST

The authors declare that there is no conflict of interest that could be perceived as prejudicing the impartiality of the research reported.

## ETHICS APPROVAL

All the animal experiments were approved by the Animal Research Ethics Committee of the University of Macau (UMARE‐022‐2019).

## AUTHOR CONTRIBUTIONS

Yu‐Lian Xu, Xiao‐Ming Jiang, and Jin‐Jian Lu designed the research; Yu‐Lian Xu, Luo‐Wei Yuan, Min‐Xia Su, and Mu‐Yang Huang performed the experiments; Yu‐Lian Xu, Xiao‐Ming Jiang, Yu‐Chi Chen, and Le‐Le Zhang, and Jin‐Jian Lu conducted the data analysis; Yu‐Lian Xu and Jin‐Jian Lu wrote the paper; Yu‐Lian Xu, Jin‐Jian Lu, Xiuping Chen, Le‐Le Zhang, and Hong Zhu revised the paper. All authors provided final approval of the paper.

## Supporting information



Supporting material

Supporting material

## Data Availability

The datasets generated and analyzed during the current study are available from the corresponding author upon reasonable request.
