# Supplementary material for: Glutathione peroxidase 8 expression on cancer cells and cancer‐associated fibroblasts facilitates lung cancer metastasis
Source: MedComm (2020). 2022 Aug 10;3(3):e152. doi: 10.1002/mco2.152 (PMC9365937; doi:10.1002/mco2.152)
Supplement: Supplementary file 1 — Supporting material [file MCO2-3-e152-s002.docx]

**Glutathione peroxidase 8 expression on cancer cells and cancer-associated fibroblasts facilitates lung cancer metastasis**

**Running title: GPX8 promotes lung cancer metastasis**

Yu-Lian Xu^1^, Luo-Wei Yuan^1^, Xiao-Ming Jiang^1^, Min-Xia Su^1^, Mu-Yang Huang^1^, Yu-Chi Chen^1^, Le-Le Zhang^1^, Xiuping Chen^1^, Hong Zhu^2^, Jin-Jian Lu^1, 3, 4, 5*^

^1^State Key Laboratory of Quality Research in Chinese Medicine, Institute of Chinese Medical Sciences, University of Macau, Macao, China

^2^Zhejiang Province Key Laboratory of Anti-Cancer Drug Research, College of Pharmaceutical Sciences, Zhejiang University, Hangzhou, China

^3^Department of Pharmaceutical Sciences, Faculty of Health Sciences, University of Macau, Macao, China

^4^MoE Frontiers Science Center for Precision Oncology, University of Macau, Macao, China

^5^Guangdong-Hong Kong-Macau Joint Lab on Chinese Medicine and Immune Disease Research, University of Macau, Macao, China

^*^Corresponding author: Dr. Jin-Jian Lu, State Key Laboratory of Quality Research in Chinese Medicine, Institute of Chinese Medical Sciences, University of Macau, Avenida da Universidade, Taipa, Macao, Email address: [jinjianlu@um.edu.mo](mailto:jinjianlu@um.edu.mo). Tel number: +853-88224674.

**Materials and Methods**

**Detection of reactive oxygen species (ROS) generation by flow cytometry**

The DCFH2-DA probe (Beyotime Biotechnology Corporation, Shanghai, China) was used to detect intracellular ROS generation. After knockdown of GPX8 for 48 h, the cells were treated with DCFH2-DA probe for 30 min and harvested in PBS. Cells pretreated with H_2_O_2_ were used as the positive control. ROS generation was conducted by flow cytometry (Becton Dickinson LSRFortessa™, Franklin Lakes, NJ, USA).

**Mutant plasmid construction and transfection**

GPX8-WT was constructed by using pCMV-Flag vector with the restriction sites of Hind III and EcoR I. Expressed protein GPX8 was fused with Flag. The C79S mutation was performed in GPX8-WT to construct the expression plasmid of GPX8-Mut. The cell suspensions (1.5 × 10^5^ cells/well) were seeded into six well-plates and cultured overnight. The cells were transfected with the indicated plasmids by using TurboFect transfection reagent (Invitrogen Corp., Carlsbad, CA, USA) for 48 h and collected for the following work.

Table S1 Primer sequence

| Primer | sequence |
| --- | --- |
| qPCR |  |
| GPX8 Forward | 5’-TACTTAGGGCTGAAGGAACTGC-3’ |
| GPX8 Reverse | 5’-GGCTCCGATTCTCCAAACTGA-3’ |
| GAPDH Forward | 5’-GCGACACCCACTCCTCCACCTTT-3’ |
| GAPDH Reverse | 5’-TGCTGTAGCCAAATTCGTTGTCATA-3’ |
| siRNA |  |
| GPX8 Sense | 5’-GGUUAGACAAGUGAUCAUATT-3’ |
| GPX8 Antisense | 5’-UAUGAUCACUUGUCUAACCTT-3’ |
| BRD2 Sense | 5’-CACUUGGCCUGCAUGACUATT-3’ |
| BRD2 Antisense | 5’-UAGUCAUGCAGGCCAAGUGTT-3’ |
| BRD3 Sense | 5’- CGGCUGAUGUUCUCGAAUUTT-3’ |
| BRD3 Antisense | 5’-AAUUCGAGAACAUCAGCCGTT-3’ |
| BRD4 Sense | 5’-GAACCUCCCUGAUUACUAUTT-3’ |
| BRD4 Antisense | 5’-AUAGUAAUCAGGGAGGUUCTT-3’ |
| shRNA |  |
| shGPX8#1 Sense | 5’-GCACTGTGTATGACTGAAATT-3’ |
| shGPX8#1 Antisense | 5’- AATTTCAGTCATACACAGTGC-3’ |
| shGPX8#3 Sense | 5’-GCCATTGCGTTTCTAATAGAA-3’ |
| shGPX8#3 Antisense | 5’- TTCTATTAGAAACGCAATGGC-3’ |
| SCR Sense | 5’-CCTAAGGTTAAGTCGCCCTCG-3’ |
| SCR Antisense | 5’-CGAGGGCGACTTAACCTTAGG-3’ |

Table S2. The RNA-seq data of A549-SCR, A549-shRNA#1, A549-shRNA#2.


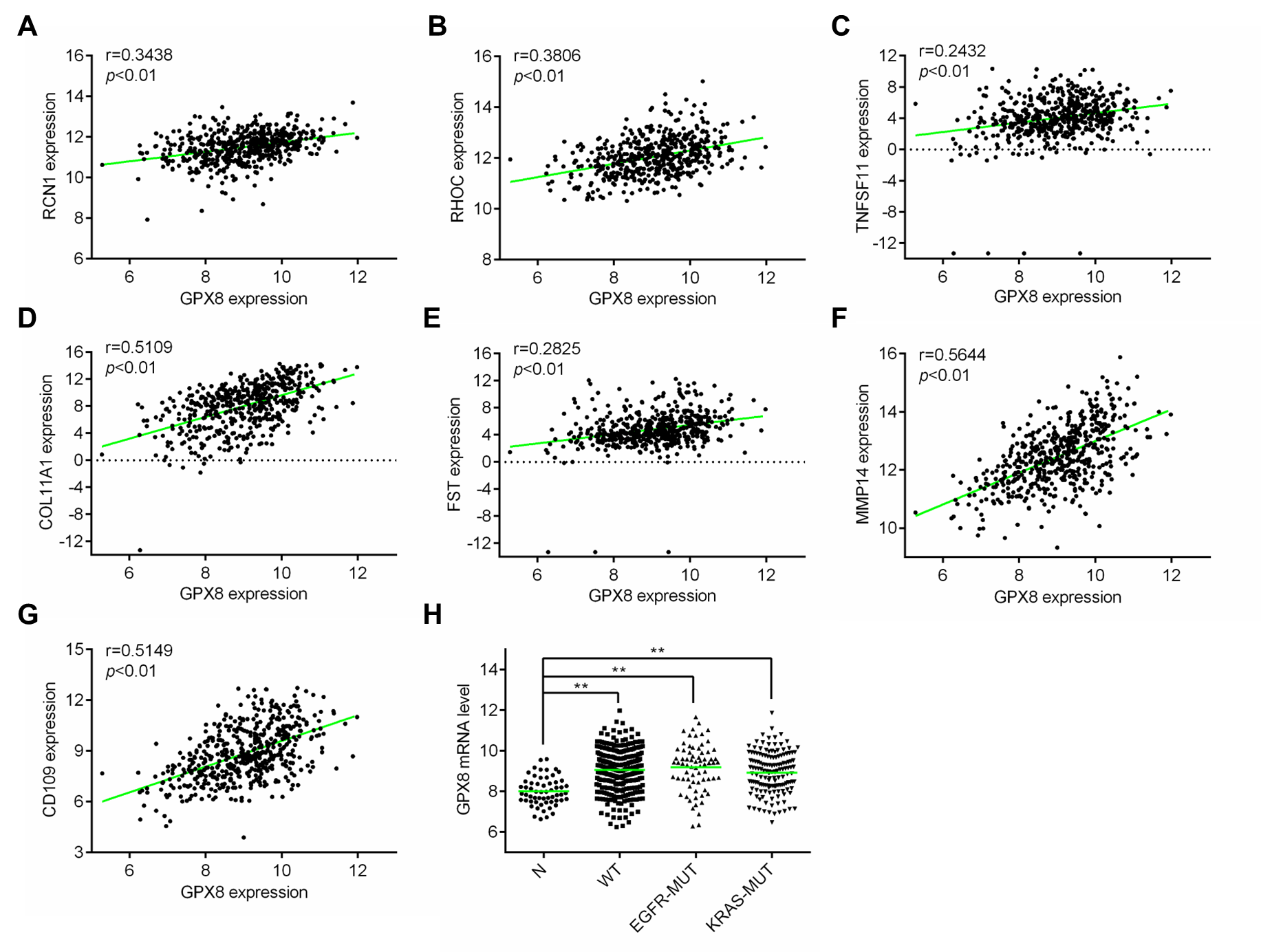


**Figure S1.** Correlation between GPX8 and RCN1 **(A)**, RHOC **(B)**, TNFSF11 **(C)**, COL11A1 **(D)**, FST **(E)**, MMP14 **(F)**, and CD109 **(G)** expression in LUAD patients from TCGA database. The *p* value is measured by Pearson’s rank correlation test. **(H)** The mRNA level of GPX8 in LUAD patients with molecular subtype EGFR and KRAS mutation. **p*<0.05 and ***p*<0.01.


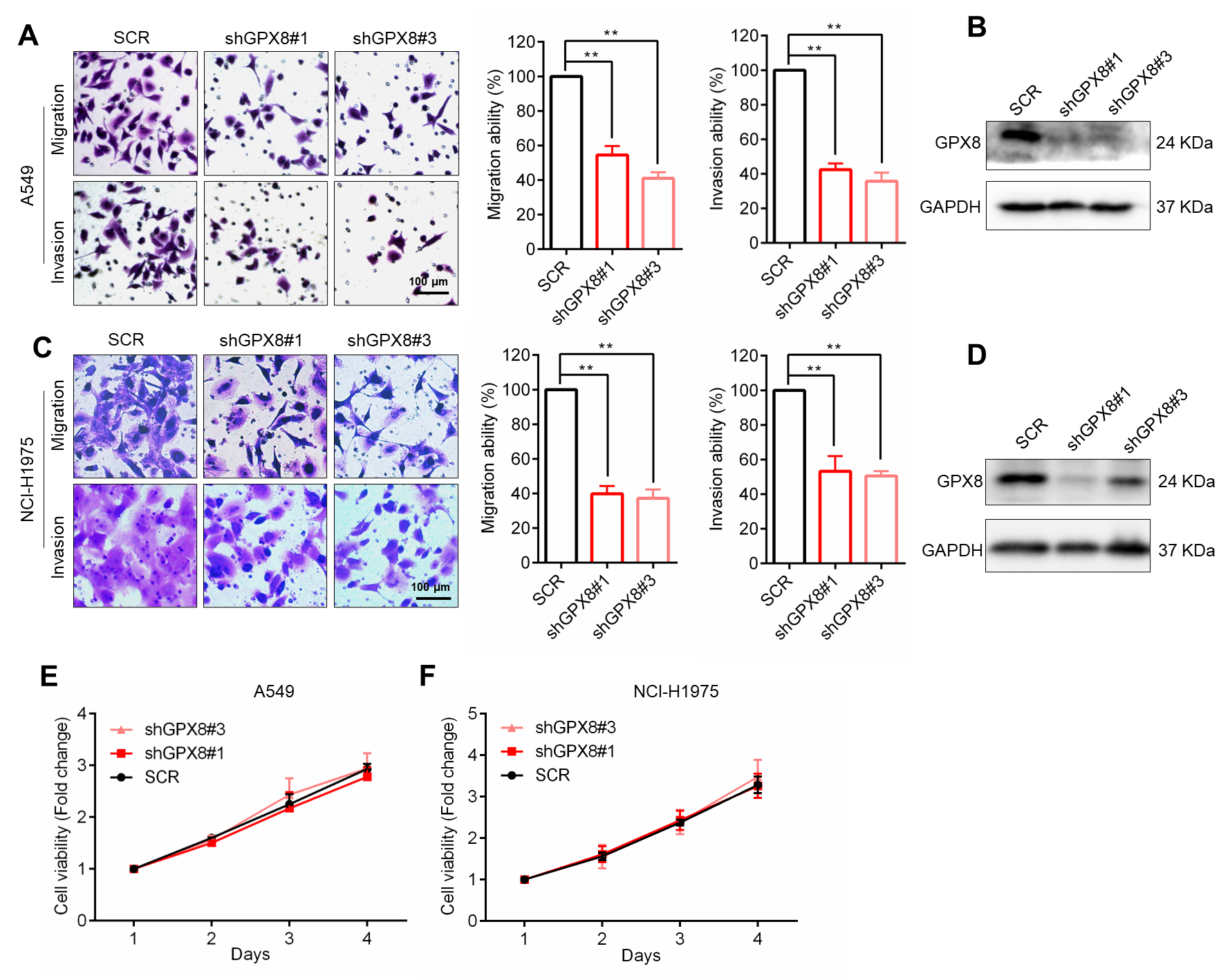


**Figure S2.** Stable knockdown of GPX8 suppressed lung cancer cell migration and invasion. **(A)** The migration/invasion abilities of A549 cells with downregulation of GPX8 by two specific shRNAs were conducted by Transwell assay. **(B)** After knockdown of GPX8 in A549 cells, the protein level of GPX8 was detected using Western blot analysis. **(C)** The migration/invasion abilities of NCI-H1975 cells with downregulation of GPX8 by two specific shRNAs were conducted by Transwell assay. **(D)** After knockdown of GPX8 in NCI-H1975 cells, the protein level of GPX8 was detected using Western blot analysis. The effects of GPX8 downregulation on A549 **(E)** and NCI-H1975 **(F)** cell growth were performed by MTT assay. **p*<0.05 and ***p*<0.01. Scale bar: 100 μm.


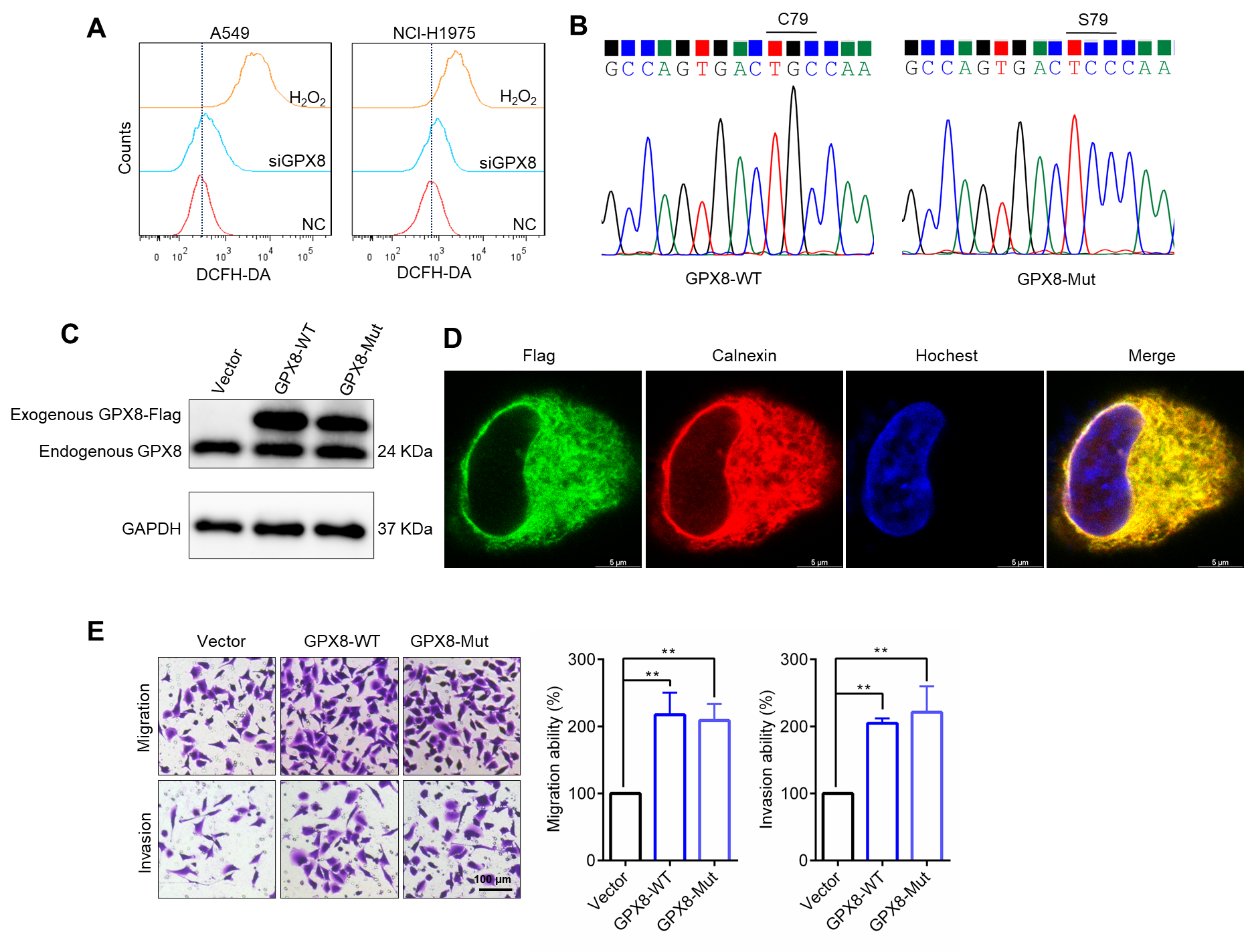


**Figure S3**. **GPX8-mediated metastasis is independent of its enzyme activity.** **(A)** After downregulation of GPX8, ROS generation was detected by flow cytometry. **(B)** Sequence of wide type GPX8 expression plasmid (GPX8-WT) and the C79S mutant GPX8 expression plasmid (GPX8-Mut). **(C)** The protein level of GPX8 was tested using Western blot analysis in A549 cells transfected with wide type GPX8 (GPX8-WT) and mutation GPX8 (GPX8-Mut). **(D)** The colocalization of Flag and Calnexin in A549 cells transfected with Flag-GPX8 was conducted using immunofluorescence. **(E)** The migration ability of A549 cells transfected with GPX8-WT and GPX8-Mut. **p*<0.05 and ***p*<0.01. Scale bar: 100 μm.


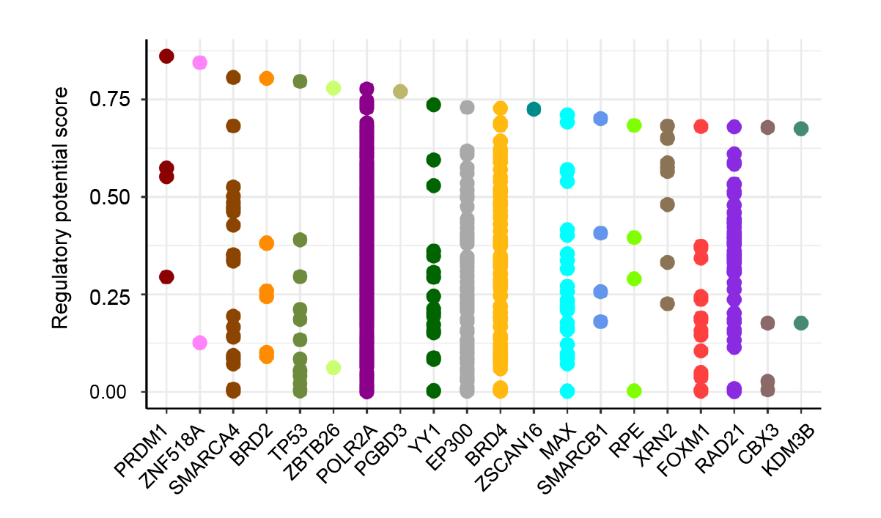


**Figure S4.** The transcription regulators for GPX8 were analyzed according to ChIP-seq data analysis by using Toolkit for Cistrome Data Browser.
